# Milk and Fermented Milk Intake and Parkinson’s Disease: Cohort Study

**DOI:** 10.3390/nu12092763

**Published:** 2020-09-10

**Authors:** Erika Olsson, Liisa Byberg, Jonas Höijer, Lena Kilander, Susanna C. Larsson

**Affiliations:** 1 Department of Surgical Sciences, Uppsala University, SE-751 85 Uppsala, Sweden; liisa.byberg@surgsci.uu.se (L.B.); Jonas.Hoijer@surgsci.uu.se (J.H.); 2 Public Health and Caring Sciences, Geriatrics, Uppsala University, SE-751 22 Uppsala, Sweden; lena.kilander@akademiska.se; 3 Unit of Cardiovascular and Nutritional Epidemiology, Institute of Environmental Medicine, Karolinska Institutet, SE-171 77 Stockholm, Sweden

**Keywords:** milk, Parkinson’s disease, risk factors

## Abstract

Milk and fermented milk consumption has been linked to health and mortality but the association with Parkinson’s disease (PD) is uncertain. We conducted a study to investigate whether milk and fermented milk intakes are associated with incident PD. This cohort study included 81,915 Swedish adults (with a mean age of 62 years) who completed a questionnaire, including questions about milk and fermented milk (soured milk and yogurt) intake, in 1997. PD cases were identified through linkage with the Swedish National Patient and Cause of Death Registers. Multivariable-adjusted hazard ratios were obtained from Cox proportional hazards regression models. During a mean follow-up of 14.9 years, 1251 PD cases were identified in the cohort. Compared with no or low milk consumption (<40 mL/day), the hazard ratios of PD across quintiles of milk intake were 1.29 (95% CI 1.07, 1.56) for 40–159 mL/day, 1.19 (95% CI 0.99, 1.42) for 160–200 mL/day, 1.29 (95% CI 1.08, 1.53) for 201–400 mL/day, and 1.14 (95% CI 0.93, 1.40) for >400 mL/day. Fermented milk intake was not associated with PD. We found a weak association between milk intake and increased risk of PD but no dose–response relationship. Fermented milk intake was not associated with increased risk of PD.

## 1. Introduction

Parkinson’s disease (PD) is the second most common neurodegenerative disease. It causes a burden not only to the afflicted individuals and their families but also to society. About four million individuals suffer from PD worldwide [1]. Currently, there are symptom-relieving medications but no cure for the disease. The pathophysiological mechanisms are not completely known but it has been suggested that inflammation, oxidative stress, and other neurotoxic processes are involved [2,3,4,5,6,7]. Therefore, identification of modifiable risk factors, such as diet, that affect the risk of PD is of great importance.

The role of diet for the development of PD is unclear, but epidemiological studies suggest that a high intake of milk may increase the risk of PD [8] (Table S1). In a meta-analysis of five prospective cohort studies [8,9,10,11,12], most with a small number of cases, the risk of PD was 56% higher among individuals in the highest versus the lowest category of milk intake [8]. In contrast, intake of fermented milk (soured milk and yogurt) has not been associated with risk of PD [10,11,12] (Table S1). However, prospective studies of fermented milk intake in relation to PD are limited and most studies were based on a small number of cases and therefore could not rule out a weak association.

To further clarify the potential associations of milk and fermented milk intake with risk of PD, we examined these associations in a north European population with a high milk intake.

## 2. Materials and Methods

### 2.1. Study Population

We obtained data from the National Research Infrastructure SIMPLER (Swedish Infrastructure for Medical Population-based Life-course Environmental Research), which includes data from the Swedish Mammography Cohort (SMC) and the Cohort of Swedish Men (COSM), which are population-based cohort studies of women and men from central Sweden (Uppsala, Västmanland, and Örebro counties). Women in the SMC were born in 1914–1948 and men in the COSM were born in 1918–1952. Participants completed detailed questionnaires about diet and other potential risk factors for chronic diseases in 1987–90 (women only), 1997, 2008, and 2009. The baseline for our study is the 1997 questionnaire (distributed to 56,030 women and 100,303 men in the late autumn of 1997). The response rate was 70% for women (*n* = 39,227) and 49% (*n* = 48,850) for men. The questionnaires were identical in the two cohorts except for some sex-specific questions.

We excluded participants with an incorrect or a missing personal identity numbers as well as those with a history of cancer or death before baseline (Figure S1). We further excluded those with an implausible energy intake (defined as 3 standard deviations from the log_e_-transformed mean energy intake in women and men separately) and a PD diagnosis before 1 January 1998 recorded in the Swedish registers (Figure S1). The final study population consisted of 81,915 participants (36,664 women and 45,271 men) aged 45–83 years.

### 2.2. Ethical Compliance Statement

The investigations were approved by the Regional Ethical Review Board in Stockholm, Sweden (Dnr: 2019-03986). All participants gave their informed consent. We confirm that we have read the Journal’s position on issues involved in ethical publication and affirm that this work is consistent with those guidelines.

### 2.3. Exposure Assessment

Dietary habits were assessed with a validated food frequency questionnaire (FFQ) [13] that captured information of average intake during the past year of 96 foods, food groups, and beverages [14]. Participants reported their average frequency (servings) of consumption of different foods. They could select from eight predefined frequency categories for most food groups (none, 1–3 times/month, 1–2 times/week, 3–4 times/week, 5–6 times/week, 1 time/day, 2 times/day, and ≥3 times/day). For milk and fermented milk, the exact numbers of glasses per day or per week could be filled in, and one glass corresponded to 200 mL. The participants could choose from three types of milk (skimmed milk (≤0.5% fat), reduced-fat milk (1.5% fat), and regular milk (3% fat or higher)) and two types of fermented milk (reduced-fat (0.5% fat) and regular (3% fat) fermented milk). The frequencies for three types of milk intake was summed as was the frequencies of the two types of fermented milk intake [15]. Energy and nutrient intakes were calculated by multiplying the reported consumption frequency of each food item with the nutrient and energy content of age-specific portion sizes obtained from the Swedish National Food Agency database [16].

The FFQ has been validated for nutrients against fourteen 24-h recall interviews in 248 Swedish men. The Spearman rank correlation coefficient was 0.65 for macronutrients and 0.62 for micronutrients [14]. For milk intake specifically, the corrected Pearson correlation coefficient was approximately 0.7 between the FFQ and four 7-day food records performed every third months [13] (A Wolk, unpublished data, 1992). Also, a positive relationship has been found between milk and fermented milk intake measured with 7-day weighted food records and 24 h recalls and the fat tissue content of pentadecanoic acid, a marker of long-term consumption of milk fat [17].

### 2.4. Ascertainment of PD

Incident and previous PD cases were identified by linkage with the Swedish National Patient (inpatient and outpatient [available from 2001]) and Cause of Death Registers. The Swedish revision of the International Classification of Diseases (ICD) codes were used for classification of PD diagnoses: 350 (ICD-7, 1964–68), 342 (ICD-8, 1969–86), 332.0 (ICD-9, 1987–96), and G20 (ICD-10, 1997–2014). The Swedish registers are valid for epidemiological studies on PD diagnosis; when comparing national registers with hospital records, the positive predictive value for PD was 70.8% and sensitivity 72.7% [18]. As outcome, we used the primary or secondary inpatient and outpatient PD diagnosis or the PD diagnosis indicated as cause of death, whichever came first.

### 2.5. Statistical Analysis

In the main analyses, time at risk of PD for each participant was calculated from baseline (1 January 1998) until the date of PD diagnosis, date of death, or end of follow-up (31 December 2014), whichever came first. Participants were categorized into approximate quintiles of glasses of milk intake per day (Q1; <40 mL/day, Q2; 40–159 mL/day, Q3; 160–200 mL/day, Q4; >201–400 mL/day, Q5; >400 mL/day). Fermented milk intake was divided into the same categories as milk intake but because of lower fermented milk intake, the two highest categories were combined. Missing information on milk and fermented milk was treated as no intake in the primary analysis [19]. Potential nonlinear trends between milk and fermented milk intake and incident PD were assessed by restricted cubic spline Cox regression with three knots placed at the 10th, 50th, and 90th percentiles of the distribution of the exposures [20].

Cox proportional hazards regression models with age as time scale was used to estimate hazard ratios with 95% confidence intervals (CI) for categories of milk and fermented milk intake. The category with the lowest intake was used as reference.

Covariates were chosen using present knowledge and directed acyclic graphs [21] and were obtained primarily from the questionnaires. Model 1 was adjusted for sex through stratification. Model 2 was adjusted for sex, smoking status, (never, former with <20 pack-years, former with ≥20 pack-years, current with <20 pack-years, or current with ≥20 pack-years), educational level (≤9 years, 10–12 years, >12 years, or other), alcohol and coffee consumption (both continuous), total energy intake (kcal/day; continuous), body mass index (weight in kg) divided by height in m^2^; continuous), physical activity (walking/bicycling and exercise during the previous year; categorical) and living alone (yes/no). Model 3 (main model) included model 2, total fruit and vegetable intake (servings/day of: apple, pear, banana, berry, orange/citrus, other fruits, carrot, beetroot, broccoli, cabbage, cauliflower, lettuce, onion, garlic, pepper, spinach, tomato, peas and pea soup, and other vegetables), vitamin and mineral supplement use (yes/no), and fermented milk (in analyses of milk) or milk intake (in analyses of fermented milk). Model 4 included model 3, aspirin use (yes/no), and self-reported high blood pressure (yes/no). An additional model (model 5) was created for women and included model 3 and postmenopausal hormone use (yes/no). Fruits and vegetables intake were included as a marker for healthy eating, and vitamin and supplement intake were included as a marker for health seeking behavior. Aspirin use was included as a confounder for its potential anti-inflammatory properties although its relation with PD is not clear-cut [22,23]. Missing information on covariates were imputed by multiple imputation using chaired equations (twenty imputations).

In a sensitivity analysis, we removed individuals with missing information on intake of milk (when milk was the exposure) and fermented milk (when fermented milk was the exposure). In an additional sensitivity analysis, we excluded the first three years of follow up through left truncation to minimize potential reverse causation bias. Finally, we performed an analysis among women using the 1987–90 investigation as baseline and time updated information on milk and soured milk intake and covariates from the 1997 investigation. All analyses were carried out in Stata 15 (StataCorp, College Station, TX, USA) using resources provided by SNIC-SENS through the Uppsala Multidisciplinary Center for Advanced Computational Science (UPPMAX).

## 3. Results

### 3.1. Characteristics of the Study Population

Baseline characteristics according to daily milk intake are shown in Table 1 for the whole study population and in Tables S2 and S3 for women and men, respectively. The mean age at baseline was 61 years. Compared with participants in the lowest category of milk intake, those in the highest category were, on average, slightly older, had a higher total energy intake and body mass index, were more likely to live alone and have lower level of education, and consumed less fruit and vegetables and vitamin and mineral supplements.

### 3.2. Associations of Milk and Fermented Milk Intake with PD

**Figure 1:**
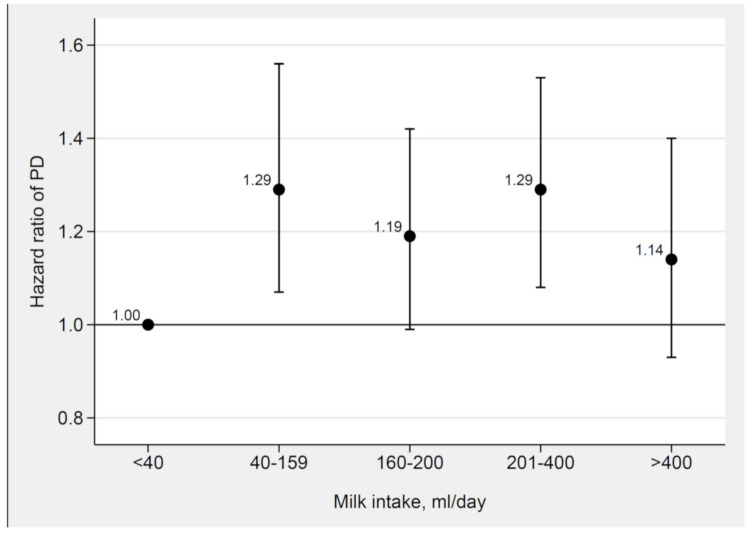
Adjusted hazard ratios for milk intake and incident Parkinson’s disease in the whole study population. Hazard ratios of the associations between milk intake and incident Parkinson’s disease adjusted for sex, smoking, education, alcohol, coffee, total energy intake, body mass index, physical activity, living alone, fruit and vegetables, vitamin and mineral supplements, and fermented milk intake using Cox proportional hazards regression with age as time scale in the whole study population (the Swedish Mammography Cohort and the Cohort of Swedish Men).

During a mean follow-up of 14.9 years (median 17 years), 1251 incident cases of PD (475 cases in women and 776 cases in men) were identified. In analyses of women and men combined, the HR of PD was higher in the four highest categories of milk intake compared with the lowest category of milk intake, with no indication of a dose–response relationship (Figure 1 and Table S4). The HRs did not change appreciably after adjustments in the different models. In the main multivariable model (model 3), the HRs of PD across categories of milk intake were 1.29 (95% CI 1.07, 1.56) for 40–159 mL/day, 1.19 (95% CI 0.99, 1.42) for 160–200 mL/day, 1.29 (95% CI 1.08, 1.53) for 201–400 mL/day, and 1.14 (95% CI 0.93, 1.40) for >400 mL/day compared with <40 mL/day. The HRs were similar in women and men but the CIs were wide and generally included the null (Tables S5 and S6).

**Figure 2:**
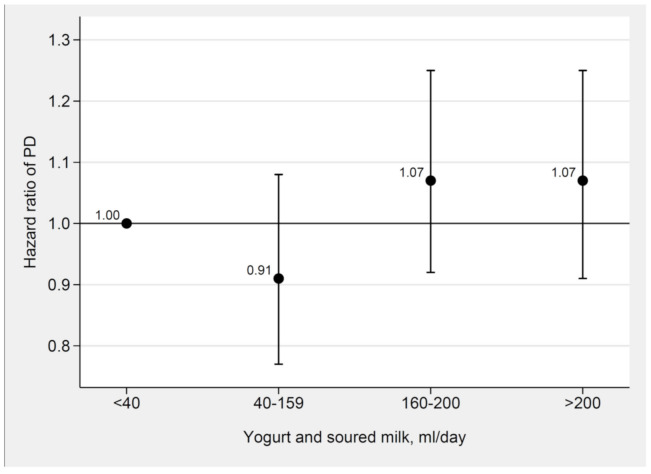
Adjusted hazard ratios for fermented milk intake and incident Parkinson’s disease in the whole study population. Hazard ratios of the associations between yogurt and soured milk intake and incident Parkinson’s disease adjusted for sex, smoking, education, alcohol, coffee, total energy intake, body mass index, physical activity, living alone, fruit and vegetables, vitamin and mineral supplements, and milk intake using Cox proportional hazard regression with age as time scale in the whole study population (the Swedish Mammography Cohort and the Cohort of Swedish Men).

There was no association between fermented milk intake and risk of PD, neither in the whole population (Figure 2 and Table S7) nor in women or men (Tables S8 and S9). These results are also illustrated as adjusted spline curves in Figures S2 and S3.

The HRs for the association between milk intake and PD were similar but with wider CIs in a sensitivity analysis excluding individuals with missing information on milk intake (Table S10). However, a similar sensitivity analysis for fermented milk showed that individuals in the highest three categories of fermented milk intake had a lower risk of PD compared with those in the lowest category (Table S11). The results for milk and fermented milk intake in relation to PD did not change materially when the first three years of follow up were excluded to evaluate whether reverse causation bias may have influenced the results (Tables S12 and S13). The HRs for the associations of time updated information on milk (Table S14) and fermented milk (Table S15) intake with PD in women were similar to those in the main analysis.

## 4. Discussion

Findings of this large cohort study of Swedish women and men indicated a weak association with milk intake and an increased risk of PD. Our finding of no association between fermented milk intake and incident PD in the primary analysis confirms the results of previous cohort studies of yogurt [10,12] and fermented milk [11] intake in relation to risk of PD (Table S1).

Our results for milk intake are broadly in line with those of previous studies from different populations of which all studies have shown that frequent milk intake is associated with an increased risk of PD in the whole study population or in women or men (Table S1). However, in contrast to most prior studies, there was no dose–response pattern between milk intake and incident PD in the present study. Specifically, the risk of PD was increased in the second category of milk intake and did not increase further with higher intakes. A possible explanation for this observation is that milk drinkers differ from nondrinkers and low consumers of milk with respect to other risk factors. Although the association remained in multivariable models adjusted for potential confounders, uncontrolled confounding cannot be ruled out. Another potential explanation is that even small amounts of milk elicit an adverse effect that increases PD risk.

As for mechanisms, a high milk intake has been associated with higher levels of oxidative stress and inflammation [24], which may play a role in the development of PD [2,3,4,5,6]. High dairy intake has also been associated with reduced serum urate levels in observational studies [25,26,27], and it has been hypothesized that urate may protect against PD through neuroprotective properties. Mendelian randomization studies have not shown a causal relationship between circulating urate levels and Parkinson’s disease [28,29,30]. Another possible mechanism is that dairy products may contain neurotoxic chemicals and pesticides that increase PD risk [7,31].

Major strengths of this study include the large sample size of men and women with dietary assessment before PD becomes manifest, especially the large number of PD cases, the use of a validated FFQ to assess dietary intake, and the objective ascertainment of PD cases. Compared to previous studies that relied on self-reported information on PD, no recall bias for PD diagnosis existed in this study owing to the objective information on PD diagnoses through data from the Swedish registers. A validation study comparing health registers and medical diagnoses from journals showed that the specificity of PD diagnoses was high in the registers [18]. Despite the high specificity, we may however not capture all incident cases of PD since the registers capture those treated in hospital with PD as main or contributing diagnosis. Thus, the association between milk intake and incident PD might reflect an association with more severe PD or PD with comorbidity. Another strength is that we had have repeated measurements of dietary intake including information on milk and fermented milk intake, as assessed in an analysis among the women in the SMC, and could capture possible changes in dietary intake during follow-up. The results from this sensitivity analysis were similar to the main results.

A potential limitation in studies of neurodegenerative diseases is that reverse causality may affect the results. A long prodromal phase with changes in olfactory function, constipation, and mild motor symptoms precedes the diagnosis of manifest PD. These subtle changes might affect dietary intake. To evaluate whether reverse causation bias may have influenced our results, we conducted a sensitivity analysis by excluding PD cases diagnosed within the first 3 years of follow-up. Results from this lag-analysis were similar to those of the main analysis. Another shortcoming is that we cannot rule out residual confounding as an explanation for the observed association between self-reported milk consumption and risk of PD. Moreover, some degree of measurement errors in the assessment of milk and fermented milk intake was inevitable and this may have affected the observed associations.

## 5. Conclusions

In conclusion, in this large cohort study, we found a weak association between milk intake and increased risk of PD but no dose-response relationship. Fermented milk intake was not associated with increased risk of PD.

## Figures and Tables

**Table 1:** Baseline characteristics of the whole study population (the Swedish Mammography Cohort and the Cohort of Swedish Men) by categories of glasses* of milk per day.

		Glasses * of Milk per Day				
Variable	Unit or Level	<0.2	0.2–0.8	0.8–1.1	1.1–2.0	>2.0
N		18,180	14,848	17,917	18,206	12,764
Age at entry	Years, mean (SD)	59.9 (9.14)	59.7 (9.01)	62.3 (9.61)	63.1 (9.72)	62.3 (9.68)
Body mass index	Kg/m^2^, mean (SD)	25.1 (3.57)	25.3 (3.57)	25.2 (3.59)	25.6 (3.67)	26.1 (3.78)
Height	Cm, mean (SD)	172 (8.86)	171 (8.8)	171 (8.81)	171 (8.9)	173 (8.79)
Weight	Kg, mean (SD)	74.2 (13.1)	74.7 (12.9)	73.9 (12.7)	75.5 (13)	78.5 (13.6)
Sex, *n* (%)	Female	8251 (45.4)	7207 (48.5)	9211 (51.4)	8042 (44.2)	3933 (30.8)
	Male	9929 (54.6)	7641 (51.5)	8706 (48.6)	10164 (55.8)	8831 (69.2)
Education, *n* (%)	<10 years	6198 (34.2)	4846 (32.7)	6723 (37.6)	7670 (42.2)	5491 (43.1)
	10–12 years	8212 (45.3)	6848 (46.2)	7824 (43.7)	7889 (43.4)	5646 (44.4)
	>12 years	3711 (20.5)	3135 (21.1)	3338 (18.7)	2603 (14.3)	1593 (12.5)
Smoking status and pack years, *n* (%)	Never smoker	7080 (39.6)	6479 (44.2)	8439 (47.9)	8388 (46.8)	5151 (41.0)
	Former <20 pack-years	4115 (23.0)	3324 (22.7)	3534 (20.1)	3207 (17.9)	2275 (18.1)
	Former ≥20 pack-years	2241 (12.5)	1507 (10.3)	1795 (10.2)	1997 (11.2)	1656 (13.2)
	Current <20	1979 (11.1)	1577 (10.8)	1840 (10.4)	1917 (10.7)	1330 (10.6)
	Current ≥20	2464 (13.8)	1755 (12.0)	2015 (11.4)	2399 (13.4)	2163 (17.2)
Walking this year, *n* (%)	Almost never	2168 (13.2)	1554 (11.3)	1872 (11.4)	2088 (12.5)	1613 (13.9)
	<20 min/day	3662 (22.4)	3072 (22.4)	3487 (21.2)	3584 (21.5)	2530 (21.8)
	20–40 min/day	5119 (31.2)	4654 (33.9)	5394 (32.8)	5144 (30.9)	3251 (28.1)
	40–60 min/day	2750 (16.8)	2301 (16.8)	2810 (17.1)	2793 (16.8)	1876 (16.2)
	>60 min/day	2683 (16.4)	2150 (15.7)	2871 (17.5)	3038 (18.2)	2318 (20.0)
Exercise this year, *n* (%)	<1 h/week	3718 (23.0)	2620 (19.5)	3100 (19.3)	3327 (20.5)	2547 (22.6)
	1 h/week	3374 (20.9)	3045 (22.6)	3457 (21.5)	3366 (20.7)	2164 (19.2)
	2–3 h/week	5049 (31.3)	4559 (33.9)	5372 (33.4)	5292 (32.6)	3443 (30.6)
	≥4 h/week	4013 (24.8)	3240 (24.1)	4174 (25.9)	4250 (26.2)	3101 (27.6)
Living alone	*N* (%)	3293 (19.3)	2513 (18.1)	3374 (20.3)	3629 (21.3)	2680 (22.0)
Aspirin use	*N* (%)	6744 (41.7)	5894 (44.0)	6812 (43.0)	6810 (42.5)	4741 (41.9)
High blood pressure	*N* (%)	3828 (21.1)	3171 (21.4)	4165 (23.2)	4582 (25.2)	3284 (25.7)
Vitamin and mineral supplements	*N* (%)	7189 (42.6)	5951 (42.5)	7319 (44.0)	6889 (41.4)	4428 (37.8)
Total energy intake	Kcal/day, mean (SD)	2130 (829)	2090 (769)	2110 (765)	2280 (810)	2730 (947)
Milk intake	Glasses/day, median (IQR)	0 (0, 0)	0.43 (0.29, 0.57)	1 (1, 1)	2 (1.71, 2)	3 (3, 4)
Fermented milk intake	Servings/day, mean (SD)	0.86 (1.05)	0.75 (0.88)	0.76 (0.88)	0.80 (1.00)	0.84 (1.23)
Coffee intake	Cups/day, mean (SD)	3.26 (2.03)	3.09 (1.86)	3.19 (1.83)	3.34 (1.89)	3.73 (2.15)
Alcohol intake	Gram/day, mean (SD)	12.3 (18.5)	11.1 (19.0)	8.59 (13.8)	8.44 (14.3)	9.55 (21.5)
Fruit and vegetable intake	Servings/day, mean (SD)	4.61 (2.85)	4.62 (2.58)	4.59 (2.69)	4.29 (2.62)	3.92 (2.51)

IQR, interquartile range; *N*, number; SD, standard deviation. * 1 glass corresponds to 200 mL.

## Supplementary Materials

The following are available online at https://www.mdpi.com/2072-6643/12/9/2763/s1, Figure S1: The Swedish Mammography Cohort (SMC) and the Cohort of Swedish Men (COSM), Figure S2: Adjusted spline curves of relation between milk intake and Parkinson’s disease in The Swedish Mammography Cohort (SMC) and the Cohort of Swedish Men (COSM), Figure S3: Adjusted spline curves of relation between fermented milk intake and Parkinson’s disease in The Swedish Mammography Cohort (SMC) and the Cohort of Swedish Men (COSM), Table S1: Characteristics of cohort studies on milk and fermented milk or yogurt consumption and Parkinson’s disease, Table S2: Baseline characteristics of women by categories of glasses* of milk per day, Table S3: Baseline characteristics of men by categories of glasses* of milk per day, Table S4: Association between milk intake and Parkinson’s disease in the whole study population, Table S5: Association between milk intake and Parkinson’s disease among women (*n* = 36,644, cases = 475), Table S6: Association between milk intake and Parkinson’s disease among men (*n* = 45,271, cases = 776), Table S7: Association between fermented milk intake and Parkinson’s disease in the whole study population, Table S8: Association between fermented milk intake and Parkinson’s disease among women (*n* = 36,644, cases = 475), Table S9: Association between fermented milk intake and Parkinson’s disease among men (*n* = 45,271, cases = 776), Table S10: Sensitivity analysis for the association of milk intake, excluding those with missing information on milk intake, with Parkinson’s disease among women and men (*n* = 71,765, cases =1124), Table S11: Sensitivity analysis for the association of fermented milk intake, excluding those with missing information on fermented milk intake, with Parkinson’s disease among women and men (*n* = 60,010, cases = 940), Table S12: Sensitivity analysis, excluding the first three years of follow-up, for the association of milk intake with Parkinson’s disease among women and men (*n* = 79,843, cases n = 1174), Table S13: Sensitivity analysis, excluding the first three years of follow-up, for the association of fermented milk intake with Parkinson’s disease among women and men (*n* = 79,843, cases = 1174), Table S14: Association between milk intake and Parkinson’s disease among women with time updated information on milk intake in 1987–89 (n = 61,424, cases n = 540, 1,405,972 person-years of follow-up), Table S15: Association between fermented milk intake and Parkinson’s disease among women with time updated information on fermented milk intake in 1987–89 (n = 61,424, cases n = 540, 1,405,972 person-years of follow-up).
