# Supplementary material for: Milk and Fermented Milk Intake and Parkinson’s Disease: Cohort Study
Source: Nutrients. 2020 Sep 10;12(9):2763. doi: 10.3390/nu12092763 (PMC7551962; doi:10.3390/nu12092763)
Supplement: Supplementary file 1 [file nutrients-12-02763-s001.pdf]

Figure S1. The Swedish Mammography Cohort (SMC) and the Cohort of Swedish Men (COSM)

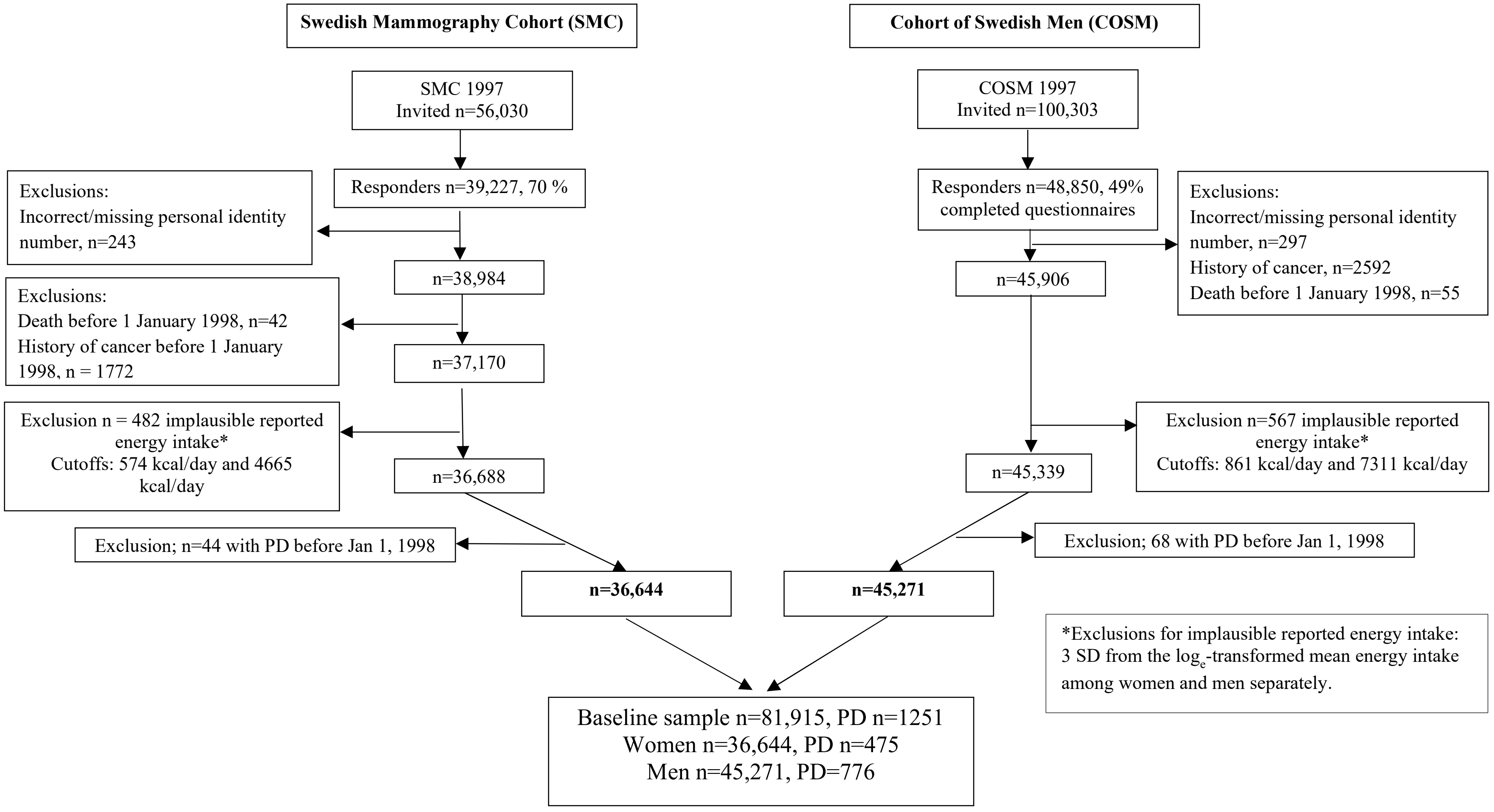

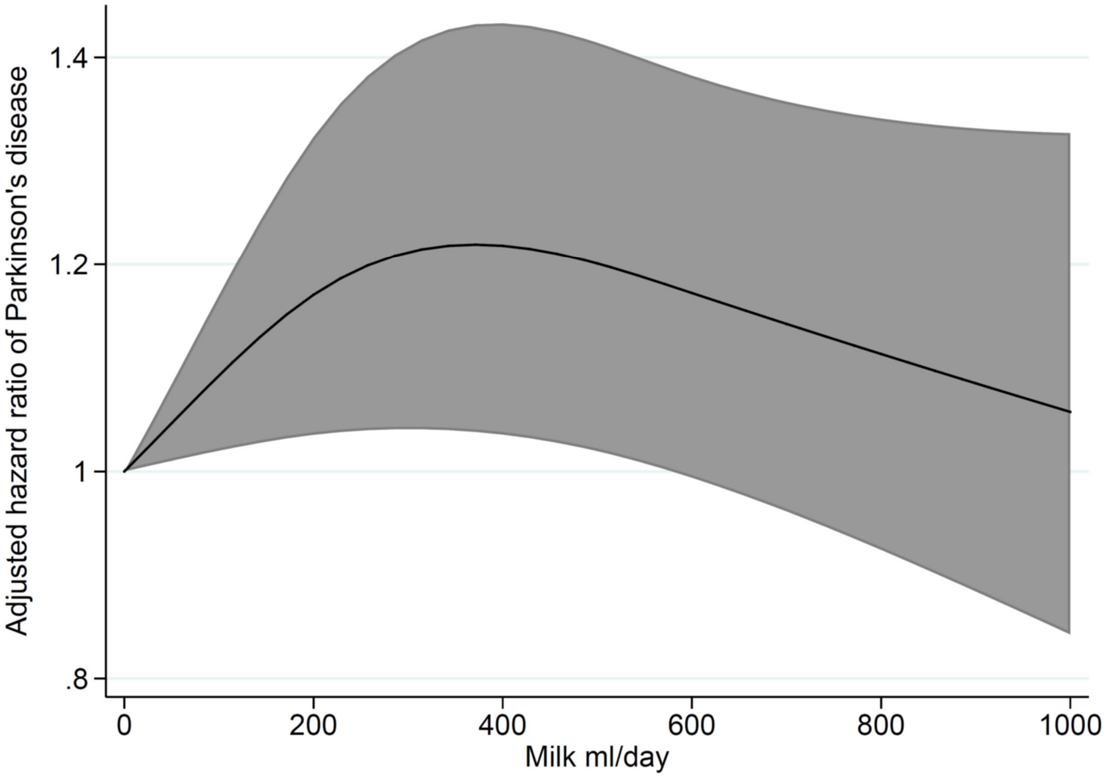

Figure S2. Adjusted spline curves of relation between milk intake and Parkinson's disease in The Swedish Mammography Cohort (SMC) and the Cohort of Swedish Men (COSM). Covariates were adjusted for sex, smoking, education, alcohol, coffee, total energy intake, body mass index, physical activity, living alone, fruit and vegetables, vitamin and mineral supplements, and fermented milk.

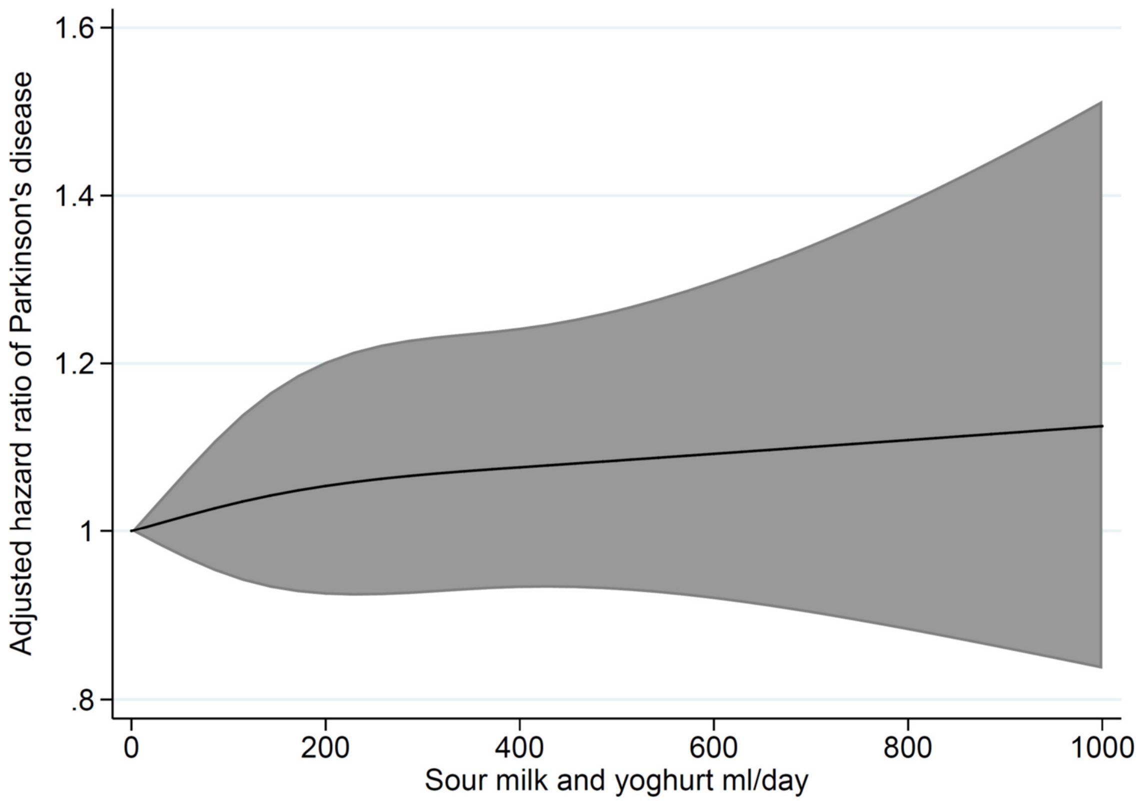

Figure S3. Adjusted spline curves of relation between fermented milk intake and Parkinson's disease in The Swedish Mammography Cohort (SMC) and the Cohort of Swedish Men (COSM). Covariates were adjusted for sex, smoking, education, alcohol, coffee, total energy intake, body mass index, physical activity, living alone, fruit and vegetables, vitamin and mineral supplements, and milk.

Table S1. Characteristics of cohort studies on milk and fermented milk or yogurt consumption and Parkinson's disease

First Author, Year	Cohort Name	Country	No. of PD Cases	Sex & no. of Participants	Age Range, Years	Follow-up years	Assessment of intake	Assessment of PD	Exposure/ Categories	RR (95% CI)	Adjustments
Park el al. (2005) [1]	Honolulu Heart Program	USA	128	M: 7504	45-68	30	24-hour recall, validated against 7-day diet record.	Prior to 1991, review of hospital records, death certificates, review of local neurologist's medical records. 1991-1993; structured interviews by technician then a neurologist. 1994 to 1996 and 1997 to 1998 participants were asked about PD diagnosis and use of PD medications. Medical records were reviewed by neurologist.	Oz/d Total milk intake:		Age, pack-years of smoking, coffee intake, physical activity, triceps skinfold, total calories and fat intake, and years worked on a plantation.
			43						0	0	
			47						>0-8	0.9 (0.6, 1.4)	
			20						8-16	1.2 (0.7, 2.0)	
			18						>16	2.3 (1.3, 4.1)	
									0	0	With additional adjustment for calcium intake.
									>0-8	1.0 (0.6, 1.5)	
									8-16	1.3 (0.7, 2.4)	
									>16	2.6 (1.1, 6.4)	
Chen et al. (2007) [2]	Cancer Prevention Study II-Nutrition Cohort from the American Cancer Society	USA	M: 250 W:138 T: 388	M: 57,689 W: 73,175 T: 130,864	50-74	9	Semi-quantitative food frequency questionnaire (FFQ).	Participants who reported a diagnosis of PD in the 2001 questionnaire were written to and asked for permission to contact their neurologist or internists. The neurologist or internists were asked to complete a diagnostic questionnaire or send a copy of the medical record. Medical records reviewed by specialist.	Quintiles (g/d)		Age, sex, smoking status, energy intake, vigorous physical activity, educational level, ibuprofen use, and pesticide exposure.
									Milk:	All	
			49						0-84.3	1.0	
			78						84.4-155.4	1.4 (1.0, 2.1)	
			87						155.4-273.1	1.6 (1.1, 2,3)	
			76						273.1-397.1	1.4 (0.9, 1.9)	
			98						>397.1	1.7 (1.2, 2.4)	
										Men	
			31						0-84.3	1.0	
			50						84.4-155.4	1.4 (0.9, 2.2)	
			54						155.4-273.1	1.5 (1.0, 2,4)	
			45						273.1-397.1	1.2 (0.7, 1.9)	
			70						>397.1	1.8 (1.2, 2.9)	
										Women	
			18						0-84.3	1.0	
			28						84.4-155.4	1.4 (0.8, 2.6)	
			33						155.4-273.1	1.8 (1.0, 3,1)	
			31						273.1-397.1	1.7 (0.9, 3.0)	
			28						>397.1	1.5 (0.8, 2.8)	
									Yogurt:	All	
			171						0	1.0	
			76						4-14.7	1.3 (1.0, 1.8)	
			44						14.7-35.4	0.9 (0.7, 1.3)	
			55						35.4-92.9	1.1 (0.8, 1.4)	
			42						>92.9	0.9 (0.6, 1.3)	
										Men	
			127						0	1.0	
			41						4-14.7	1.2 (0.8, 1.7)	
			27						14.7-35.4	1.0 (0.7, 1.5)	

			33<br>22<br><br>44<br>35<br>17<br>22<br>20						35.4-92.9<br>>92.9<br><br>0<br>4-14.7<br>14.7-35.4<br>35.4-92.9<br>>92.9	1.1 (0.7, 1.5)<br>0.9 (0.6, 1.4)<br>Women<br>1.0<br>1.7 (1.1, 2.6)<br>0.9 (0.5, 1.6)<br>1.1 (0.7, 1.8)<br>1.0 (0.6, 1.6)	
Sääksjärvi et al. (2013) [3]	Finnish Mobile Clinic Survey	Finland	M: 45<br>W: 40<br>T: 85<br><br>10<br>21<br>14<br><br>5<br>16<br>19<br><br><br><br><br><br><br>12<br>17<br>16<br><br>11<br>18<br>11	M: 2388<br>W: 2136<br>T: 4524	40-79	41	Dietary history interview, food item per day, week, month, year, and portion sizes included, trained interviewers.	PD-cases identified through linkage with Drug Imbursement Register of the Social Insurance Institution. All individuals in Finland with PD are eligible for medication free of charge and must have a certificate from a treating neurologist. Re-evaluated by the study neurologist.	Tertiles (g/d)<br><br><br>Milk:<br>T1≤ 545<br>T2≤ 950<br>T3> 950<br><br>T1≤ 370<br>T2≤ 613<br>T3> 613<br>Milk:<br><br>Highest vs. lowest tertile<br>Fermented milk:<br>T1< 1<br>T2≤ 150<br>T3> 150<br><br>T1≤ 23<br>T2≤ 166<br>T3> 166	 <br><br>Men<br>1.00<br>1.99 (0.91, 4.38)<br>1.24 (0.48, 3.17)<br>Women<br>1.00<br>3.08 (1.10, 8.60)<br>3.31 (1.10, 9.93)<br>Combined men and women<br>2.16 (1.09, 4.28)<br><br>Men<br><br>1.00<br>1.45 (0.68, 3.10)<br>1.71 (0.79, 3.70)<br>Women<br>1.00<br>1.48 (0.69, 3.16)<br>0.76 (0.32, 1.80)	Age, sex, marital status, community density, geographical area, smoking, BMI, leisure-time physical activity, energy, hypertension, serum cholesterol, diabetes, and in women parity.
Kyrozis et al. (2013) [4]	The European Prospective Investigation into Cancer and Nutrition (EPIC) study	Greece	M:37<br>W:51<br>T: 88	M:10,344<br>W:15,063<br>T: 25,407	20-86		Semi-quantitative questionnaire, trained interviewers.	Participants were asked to report medical diagnosis of PD or use of anti-PD drugs. Possible incident cases were assessed by telephone interview.	Effect estimates for a 1-SD increase in intake (g/d)<br><br>Milk<br>Yogurt	 <br><br><br><br>All<br>1.34 (1.14, 1.58)<br>0.86 (0.65, 1.15)	Gender, age, marital status, education, farming, smoking, coffee with caffeine, BMI, physical activity, and energy intake.
Hughes et al (2017) [5]	Health Professionals Follow-up Study and Nurses' Health Study	USA	M: 554<br>W: 482<br>T: 1036<br><br>53<br>134	M: 48,610<br>W: 80,736<br>T: 129,346	M:40-75<br>W:30-55	M: 24<br>W: 26	FFQ	PD cases were identified with biennial self-report questionnaires. Before 2003, those who indicated PD diagnosis, their neurologist were contacted or medical records were reviewed. Since 2003 all who self-reported	Servings per week or day<br><br><br>Total milk:<br>≤ 3/month<br>1-4/week	 <br><br>Men<br>1.0<br>1.41 (1.02, 1.95)	Age, pack-years of smoking, coffee intake, body mass index, physical activity, alcohol intake, and total energy intake.

177	PD, their records were reviewed	5/week-1/day	1.23 (0.90, 1.68)
190	by a neurologist.	> 1/day	1.42 (1.03, 1.96)
			Women
34		≤ 3/month	1.0
99		1-4/week	1.18 (0.79, 1.75)
152		5/week-1/day	1.08 (0.74, 1.57)
197		> 1/day	1.30 (0.88, 1.90)
		Total milk:	Pooled analysis men and women
		≤ 3/month	1.0
		1-4/week	1.31 (1.02, 1.69)
		5/week-1/day	1.16 (0.91, 1.48)
		> 1/day	1.37 (1.07, 1.75)

Abbreviations: BMI, Body Mass Index; CI, confidence interval; d, day; FFQ, food frequency questionnaire; M, Men; No, Numbers; PD, Parkinson's disease; SD, standard deviation; T, Total population; W, Women.

1. Park, M.; Ross, G.W.; Petrovitch, H.; White, L.R.; Masaki, K.H.; Nelson, J.S.; Tanner, C.M.; Curb, J.D.; Blanchette, P.L.; Abbott, R.D. Consumption of milk and calcium in midlife and the future risk of Parkinson disease. *Neurology* **2005**, *64*, 1047-1051, doi:10.1212/01.WNL.0000154532.98495.BF.
2. Chen, H.; O'Reilly, E.; McCullough, M.L.; Rodriguez, C.; Schwarzschild, M.A.; Calle, E.E.; Thun, M.J.; Ascherio, A. Consumption of dairy products and risk of Parkinson's disease. *Am. J. Epidemiol.* **2007**, *165*, 998-1006, doi:10.1093/aje/kwk089.
3. Saaksjarvi, K.; Knekt, P.; Lundqvist, A.; Mannisto, S.; Heliovaara, M.; Rissanen, H.; Jarvinen, R. A cohort study on diet and the risk of Parkinson's disease: the role of food groups and diet quality. *Br. J. Nutr.* **2013**, *109*, 329-337, doi:10.1017/S0007114512000955.
4. Kyrozis, A.; Ghika, A.; Stathopoulos, P.; Vassilopoulos, D.; Trichopoulos, D.; Trichopoulou, A. Dietary and lifestyle variables in relation to incidence of Parkinson's disease in Greece. *Eur. J. Epidemiol.* **2013**, *28*, 67-77, doi:10.1007/s10654-012-9760-0.
5. Hughes, K.C.; Gao, X.; Kim, I.Y.; Wang, M.; Weisskopf, M.G.; Schwarzschild, M.A.; Ascherio, A. Intake of dairy foods and risk of Parkinson disease. *Neurology* **2017**, *89*, 46-52, doi:10.1212/WNL.0000000000004057.

Table S2. Baseline characteristics of women by categories of glasses* of milk per day

		**Glasses* of milk per day**				
**Factor**	**Unit or Level**	**<0.2**	**0.2-0.8**	**0.8-1.1**	**1.1-2.0**	**>2.0**
N		8251	7207	9211	8042	3933
Age at entry	Years, mean (SD)	60.4 (8.81)	60.5 (8.61)	63 (9.32)	64.2 (9.47)	64.2 (9.26)
Body Mass Index	Kg/m^2, mean (SD)	24.5 (3.81)	25 (3.87)	25 (3.86)	25.4 (4.03)	25.5 (4.17)
Height	Cm, mean (SD)	165 (5.72)	165 (5.69)	165 (5.74)	164 (5.85)	164 (5.83)

Weight	Kg, mean (SD)	66.7 (10.9)	68.1 (11.2)	67.8 (11.1)	68.6 (11.4)	69.1 (11.8)
Education, n (%)	<10 years	3077 (37.4)	2658 (36.9)	3869 (42.0)	3768 (46.9)	1844 (46.9)
	10-12 years	3312 (40.2)	2967 (41.2)	3545 (38.5)	3061 (38.1)	1492 (37.9)
	>12 years	1846 (22.4)	1579 (21.9)	1791 (19.5)	1207 (15.0)	596 (15.2)
Smoking status and pack years, n (%)	Never smoker	3849 (47.6)	3687 (52.0)	5118 (56.6)	4569 (57.9)	2159 (55.7)
	Ex <20 pack-years	1699 (21.0)	1442 (20.3)	1522 (16.8)	1134 (14.4)	531 (13.7)
	Ex >=20 pack-years	517 (6.4)	363 (5.1)	471 (5.2)	390 (4.9)	190 (4.9)
	Current <20	1074 (13.3)	910 (12.8)	1122 (12.4)	982 (12.4)	491 (12.7)
	Current >=20	941 (11.6)	691 (9.7)	805 (8.9)	822 (10.4)	502 (13.0)
Walk this year, n (%)	Almost never	867 (11.6)	641 (9.5)	867 (10.2)	861 (11.6)	478 (13.3)
	<20 min/day	1465 (19.5)	1274 (18.9)	1601 (18.8)	1399 (18.9)	642 (17.8)
	20-40 min/day	2531 (33.7)	2480 (36.8)	2986 (35.1)	2466 (33.3)	1130 (31.4)
	40-60 min/day	1384 (18.4)	1262 (18.7)	1563 (18.4)	1377 (18.6)	669 (18.6)
	>60 min/day	1255 (16.7)	1079 (16.0)	1484 (17.5)	1295 (17.5)	680 (18.9)
Exercise this year, n (%)	<1 hour/week	1595 (21.7)	1164 (17.8)	1571 (19.0)	1398 (19.7)	721 (20.9)
	1 hour/week	1704 (23.2)	1651 (25.2)	1961 (23.7)	1660 (23.3)	752 (21.8)
	2-3 hours/week	2361 (32.2)	2307 (35.2)	2832 (34.2)	2429 (34.1)	1119 (32.5)
	>=4 hours/week	1681 (22.9)	1434 (21.9)	1909 (23.1)	1627 (22.9)	855 (24.8)
Living alone	N, (%)	1560 (21.8)	1367 (21.9)	1961 (24.6)	1850 (26.5)	905 (26.7)
Aspirin use	N, (%)	3599 (50.3)	3229 (51.2)	3972 (50.1)	3466 (50.5)	1769 (52.4)
High blood pressure	N, (%)	1482 (18.0)	1429 (19.8)	1983 (21.5)	1901 (23.6)	931 (23.7)
Hormone replacement	N, (%)	4254 (51.6)	3708 (51.4)	4503 (48.9)	3724 (46.3)	1830 (46.5)
Vitamin- and mineral supplements	N, (%)	4177 (54.5)	3655 (53.9)	4568 (53.6)	3912 (53.4)	1932 (53.6)
Total energy intake	Kcal/day, mean (SD)	1648 (524)	1661 (491)	1698 (494)	1794 (514)	2025 (585)
Fermented milk intake	Servings/day, mean (SD)	0.914 (1.03)	0.805 (0.88)	0.83 (0.89)	0.883 (1.01)	1 (1.39)
Coffee intake	Cups/day, mean (SD)	3.04 (1.81)	2.93 (1.68)	3.05 (1.7)	3.16 (1.75)	3.44 (1.9)
Alcohol intake	Gram/day, mean (SD)	6.52 (8.8)	6.6 (10.4)	4.89 (7.03)	4.3 (6.69)	4.51 (15.1)
Fruit and vegetables	Servings/day, mean (SD)	5.25 (3.02)	5.22 (2.72)	5.13 (2.87)	4.91 (2.86)	4.78 (2.77)
Milk intake	Glasses/day, median (IQR)	0 (0, 0)	0.43 (0.29, 0.57)	1 (1, 1)	2 (1.86, 2)	3 (3, 4)

IQR, interquartile range; N, number; SD, standard deviation

*1 glass corresponds to 200 ml.

Table S3. Baseline characteristics of men by categories of glasses* of milk per day

		Glasses* of milk per day				
**Factor**	**Unit or level**	**<0.2**	**0.2-0.8**	**0.8-1.1**	**1.1-2.0**	**>2.0**
N		9929	7641	8706	10164	8831
Age at entry	Years, mean (SD)	59.5 (9.38)	59 (9.32)	61.6 (9.86)	62.2 (9.82)	61.4 (9.75)
Body Mass Index	Kg/m^2, mean (SD)	25.6 (3.28)	25.7 (3.22)	25.5 (3.24)	25.8 (3.33)	26.3 (3.55)
Height	Cm, mean (SD)	177 (6.74)	178 (6.67)	177 (6.62)	177 (6.64)	177 (6.79)
Weight	Kg, mean (SD)	80.4 (11.4)	80.8 (11.2)	80.2 (11.1)	80.9 (11.5)	82.6 (12.2)
Education, n (%)	<10 years	3121 (31.6)	2188 (28.7)	2854 (32.9)	3902 (38.5)	3647 (41.5)
	10-12 years	4900 (49.6)	3881 (50.9)	4279 (49.3)	4828 (47.7)	4154 (47.2)
	>12 years	1865 (18.9)	1556 (20.4)	1547 (17.8)	1396 (13.8)	997 (11.3)
Smoking status and pack years, n (%)	Never smoker	3231 (33.0)	2792 (37.0)	3321 (38.7)	3819 (38.1)	2992 (34.4)
	Ex <20 pack-years	2416 (24.7)	1882 (24.9)	2012 (23.4)	2073 (20.7)	1744 (20.0)
	Ex >=20 pack-years	1724 (17.6)	1144 (15.2)	1324 (15.4)	1607 (16.1)	1466 (16.8)
	Current <20	905 (9.2)	667 (8.8)	718 (8.4)	935 (9.3)	839 (9.6)
	Current >=20	1523 (15.5)	1064 (14.1)	1210 (14.1)	1577 (15.8)	1661 (19.1)
Walk this year, n (%)	Almost never	1301 (14.7)	913 (13.1)	1005 (12.7)	1227 (13.3)	1135 (14.2)
	<20 min/day	2197 (24.7)	1798 (25.7)	1886 (23.8)	2185 (23.6)	1888 (23.6)
	20-40 min/day	2588 (29.1)	2174 (31.1)	2408 (30.4)	2678 (29.0)	2121 (26.5)
	40-60 min/day	1366 (15.4)	1039 (14.9)	1247 (15.7)	1416 (15.3)	1207 (15.1)
	>60 min/day	1428 (16.1)	1071 (15.3)	1387 (17.5)	1743 (18.8)	1638 (20.5)
Exercise this year, n (%)	<1 hour/week	2123 (24.1)	1456 (21.1)	1529 (19.5)	1929 (21.1)	1826 (23.4)
	1 hour/week	1670 (18.9)	1394 (20.2)	1496 (19.1)	1706 (18.7)	1412 (18.1)
	2-3 hours/week	2688 (30.5)	2252 (32.6)	2540 (32.4)	2863 (31.4)	2324 (29.8)
	>=4 hours/week	2332 (26.5)	1806 (26.1)	2265 (28.9)	2623 (28.8)	2246 (28.8)
Living alone	N, (%)	1733 (17.5)	1146 (15.0)	1413 (16.3)	1779 (17.6)	1775 (20.2)
Aspirin use	N, (%)	3145 (34.8)	2665 (37.7)	2840 (35.9)	3344 (36.4)	2972 (37.5)
High blood pressure	N, (%)	2346 (23.6)	1742 (22.8)	2182 (25.1)	2681 (26.4)	2353 (26.6)
Vitamin- and mineral supplements	N, (%)	3012 (32.7)	2296 (31.7)	2751 (33.9)	2977 (31.9)	2496 (30.7)
Total energy intake	Kcal/day, mean (SD)	2529 (824)	2503 (760)	2547 (759)	2668 (793)	3044 (908)
Fermented milk intake	Servings/day, mean (SD)	0.815 (1.06)	0.704 (0.867)	0.682 (0.857)	0.727 (0.978)	0.772 (1.15)
Coffee intake	Cups/day, mean (SD)	3.44 (2.17)	3.25 (2.01)	3.33 (1.95)	3.48 (1.99)	3.87 (2.24)

Alcohol intake	Gram/day, mean (SD)	17 (22.6)	15.4 (23.7)	12.5 (17.6)	11.7 (17.5)	11.8 (23.5)
Fruit and vegetables	Servings/day, mean (SD)	4.07 (2.58)	4.06 (2.3)	4.02 (2.35)	3.81 (2.29)	3.54 (2.28)
Milk intake	Glasses/day, median (IQR)	0 (0, 0)	0.43 (0.29, 0.71)	1 (1, 1)	2 (1.71, 2)	3 (3, 4)

IQR, interquartile range; N, number; SD, standard deviation

*1 glass corresponds to 200 ml.

Table S4. Association between milk intake and Parkinson’s disease in the whole study population

Glasses* per day (median intake in glasses* per day)	Total N	Cases	Model 1[1,2] HR (95% CI)	Model 2[3,4] HR (95% CI)	Model 3[5,6] HR (95% CI)	Model 4[7,8] HR (95% CI)
<0.2 (0)	18 180	214	Referent	Referent	Referent	Referent
0.2-0.8 (0.43)	14 848	224	1.29 (1.07, 1.55)	1.26 (1.05, 1.53)	1.29 (1.07, 1.56)	1.29 (1.07, 1.56)
0.8-1.1 (1)	17 917	283	1.22 (1.02, 1.45)	1.18 (0.99, 1.42)	1.19 (0.99, 1.42)	1.19 (0.99, 1.42)
1.1-2.0 (2)	18 206	326	1.31 (1.10, 1.56)	1.29 (1.08, 1.54)	1.29 (1.08, 1.53)	1.29 (1.08, 1.54)
>2.0 (3)	12 764	204	1.16 (0.96, 1.41)	1.16 (0.95, 1.42)	1.14 (0.93, 1.40)	1.15 (0.94, 1.41)

CI, confidence interval; HR, hazard ratio; N, number

*1 glass = 200 ml.

[1]Adjusted for sex.

[2]P-value for the combined test of the different categories: 0.027.

[3]Adjusted for sex, smoking, education, alcohol, coffee, total energy intake, body mass index, physical activity, living alone.

[4]P-value for the combined test of the different categories: 0.054.

[5]Adjusted for sex, smoking, education, alcohol, coffee, total energy intake, body mass index, physical activity, living alone, fruit and vegetables, vitamin and mineral supplements, and fermented milk.

[6]P-value for the combined test of the different categories: 0.041.

[7]Adjusted for sex, smoking, education, alcohol, coffee, total energy intake, body mass index, physical activity, living alone, fruit and vegetables, vitamin and mineral supplements, aspirin, fermented milk, and high blood pressure.

[8]P-value for the combined test of the different categories: 0.039.

Table S5. Association between milk intake and Parkinson’s disease among women (n=36 644, cases=475)

Glasses* per day	Total N	Cases	Mode l[1,2] HR (95% CI)	Model 2[3,4] HR (95% CI)	Model 3[5,6] HR (95% CI)	Model 4[7,8] HR (95% CI)	Model 5[9,10] HR (95% CI)
<0.2	8251	77	Referent	Referent	Referent	Referent	Referent
0.2-0.8	7207	94	1.36 (1.01, 1.84)	1.33 (0.98, 1.80)	1.35 (1.00, 1.83)	1.36 (1.00, 1.84)	1.35 (1.00, 1.83)

0.8-1.1	9211	120	1.22 (0.92, 1.63)	1.18 (0.88, 1.57)	1.18 (0.88, 1.57)	1.18 (0.88, 1.58)	1.18 (0.88, 1.57)
1.1-2.0	8042	122	1.37 (1.03, 1.83)	1.32 (0.99, 1.77)	1.29 (0.96, 1.72)	1.30 (0.97, 1.74)	1.29 (0.96, 1.73)
>2.0	3933	62	1.41 (1.01, 1.98)	1.33 (0.94, 1.88)	1.27 (0.89, 1.80)	1.28 (0.90, 1.82)	1.27 (0.89, 1.80)

CI, confidence interval; HR, hazard ratio; N, number

*1 glass = 200 ml.

[1]Crude model.

[2]P-value for the combined test of the different categories: 0.169.

[3]Adjusted for smoking, education, alcohol, coffee, total energy intake, body mass index, physical activity, living alone

[4]P-value for the combined test of the different categories: 0.293.

[5]Adjusted for smoking, education, alcohol, coffee, total energy intake, body mass index, physical activity, living alone, fruit and vegetables, vitamin and mineral supplements, yogurt and soured milk.

[6]P-value for the combined test of the different categories: 0.347.

[7]Adjusted for smoking, education, alcohol, coffee, total energy intake, body mass index, physical activity, living alone, fruit and vegetables, vitamin and mineral supplements, aspirin, yogurt and soured milk, and high blood pressure

[8]P-value for the combined test of the different categories: 0.329.

[9]Adjusted for smoking, education, alcohol, coffee, total energy intake, body mass index, physical activity, living alone, fruit and vegetables, vitamin and mineral supplements, aspirin, yogurt and soured milk, high blood pressure and hormone replacement.

[10]P-value for the combined test of the different categories: 0.345.

Table S6. Association between milk intake and Parkinson's disease among men (n=45 271, cases=776)

Glasses* per day	Total N	Cases	Model 1[1,2] Hazard ratio (95 % CI)	Model 2[3,4] Hazard ratio (95 % CI)	Model 3[5,6] Hazard ratio (95 % CI)	Model 4[7,8] Hazard ratio (95 % CI)
<0.2	9929	137	Referent	Referent	Referent	Referent
0.2-0.8	7641	130	1.24 (0.98, 1.58)	1.22 (0.96, 1.55)	1.25 (0.98, 1.59)	1.25 (0.98, 1.59)
0.8-1.1	8706	163	1.22 (0.97, 1.53)	1.20 (0.95, 1.51)	1.20 (0.95, 1.51)	1.20 (0.95, 1.51)
1.1-2.0	10164	204	1.28 (1.03, 1.59)	1.28 (1.03, 1.60)	1.29 (1.03, 1.61)	1.29 (1.03, 1.61)
>2.0	8831	142	1.06 (0.84, 1.34)	1.10 (0.86, 1.41)	1.10 (0.86, 1.41)	1.10 (0.86, 1.42)

CI, confidence interval; HR, hazard ratio; N, number

*1 glass = 200 ml.

[1]Crude model.

[2]P-value for the combined test of the different categories: 0.139

[3]Adjusted for smoking, education, alcohol, coffee, total energy intake, body mass index, physical activity, living alone

[4]P-value for the combined test of the different categories: 0.223

[5]Adjusted for smoking, education, alcohol, coffee, total energy intake, body mass index, physical activity, living alone, fruit and vegetables, vitamin and mineral supplements, yogurt and soured milk

[6]P-value for the combined test of the different categories: 0.181

[7]Adjusted for smoking, education, alcohol, coffee, total energy intake, body mass index, physical activity, living alone, fruit and vegetables, vitamin and mineral supplements, aspirin, yogurt and soured milk, and high blood pressure

[8]P-value for the combined test of the different categories: 0.178

Table S7. Association between fermented milk intake and Parkinson's disease in the whole study population

Glasses* per day (median intake in glasses* per day)	Total N	Cases	Model 1[1,2] HR (95% CI)	Model 2[3,4] HR (95% CI)	Model 3[5,6] HR (95% CI)	Model 4[7,8] HR (95% CI)
<0.2 (0)	29007	428	Referent	Referent	Referent	Referent
0.2-0.8 (0.43)	16475	218	0.95 (0.81, 1.12)	0.92 (0.78, 1.09)	0.91 (0.77, 1.08)	0.91 (0.77, 1.08)
0.8-1.1 (1)	19687	325	1.11 (0.96, 1.29)	1.06 (0.92, 1.23)	1.07 (0.92, 1.25)	1.08 (0.93, 1.25)
>1.1 (2)	16746	280	1.09 (0.94, 1.27)	1.06 (0.91, 1.24)	1.07 (0.91, 1.25)	1.07 (0.91, 1.25)

CI, confidence interval; HR, hazard ratio; N, number

*1 glass = 200 ml.

[1]Adjusted for sex.

[2] P-value for the combined test of the different categories: 0.218.

[3]Adjusted for sex, smoking, education, alcohol, coffee, total energy intake, body mass index, physical activity, living alone.

[4] P-value for the combined test of the different categories: 0.371.

[5]Adjusted for sex, smoking, education, alcohol, coffee, total energy intake, body mass index, physical activity, living alone, fruit and vegetables, vitamin- and mineral supplements, and milk.

[6] P-value for the combined test of the different categories: 0.244.

[7]Adjusted for sex, smoking, education, alcohol, coffee, total energy intake, body mass index, physical activity, living alone, fruit and vegetables, vitamin and mineral supplements, aspirin, milk, and high blood pressure.

[8] P-value for the combined test of the different categories: 0.247.

Table S8. Association between fermented milk intake and Parkinson's disease among women (n=36 644, cases=475)

Glasses* per day	Total N	Cases	Model 1[1,2] HR (95 % CI)	Model 2[3,4] HR (95 % CI)	Model 3[5,6] HR (95 % CI)	Model 4[7,8] HR (95 % CI)	Model 5[9,10] HR (95 % CI)
<0.2	10134	124	Referent	Referent	Referent	Referent	Referent

0.2-0.8	8229	96	1.02 (0.78, 1.33)	0.99 (0.76, 1.30)	0.99 (0.75, 1.30)	0.99 (0.75, 1.30)	0.98 (0.75, 1.29)
0.8-1.1	10699	143	1.05 (0.83, 1.34)	1.01 (0.79, 1.29)	1.04 (0.81, 1.34)	1.04 (0.81, 1.34)	1.04 (0.81, 1.33)
>1.1	7582	112	1.16 (0.90,1.49)	1.09 (0.83, 1.43)	1.11 (0.84, 1.45)	1.11 (0.84, 1.45)	1.10 (0.84, 1.44)

CI, confidence interval; HR, hazard ratio; N, number

*1 glass = 200 ml.

[1]Crude model.

[2]P-value for the combined test of the different categories: 0.703

[3]Adjusted for smoking, education, alcohol, coffee, total energy intake, body mass index, physical activity, living alone

[4]P-value for the combined test of the different categories: 0.891

[5]Adjusted for smoking, education, alcohol, coffee, total energy intake, body mass index, physical activity, living alone, fruit and vegetables, vitamin and mineral supplements, and milk

[6]P-value for the combined test of the different categories: 0.853

[7]Adjusted for smoking, education, alcohol, coffee, total energy intake, body mass index, physical activity, living alone, fruit and vegetables, vitamin and mineral supplements, aspirin, milk, and high blood pressure

[8]P-value for the combined test of the different categories: 0.857

[9]Adjusted for smoking, education, alcohol, coffee, total energy intake, body mass index, physical activity, living alone, fruit and vegetables, vitamin and mineral supplements, aspirin, milk, high blood pressure and hormone replacement

[10]P-value for the combined test of the different categories: 0.876

Table S9. Association between fermented milk intake and Parkinson's disease among men (n=45 271, cases= 776)

Glasses* per day	Total N	Cases	Model 1[1,2] HR (95% CI)	Model 2[3,4] HR (95% CI)	Model 3[5,6] HR (95% CI)	Model 4[7,8] HR (95% CI)
<0.2	18873	304	Referent	Referent	Referent	Referent
0.2-0.8	8246	122	0.91 (0.74, 1.12)	0.88 (0.71, 1.09)	0.87 (0.70 1.07)	0.87 (0.70, 1.07)
0.8-1.1	8988	182	1.17 (0.97, 1.40)	1.11 (0.92, 1.34)	1.11 (0.92, 1.34)	1.11 (0.92, 1.34)
>1.1	9164	168	1.06 (0.88, 1.28)	1.03 (0.85, 1.25)	1.03 (0.85, 1.25)	1.03 (0.84, 1.25)

CI, confidence interval; HR, hazard ratio; N, number

*1 glass = 200 ml.

[1]Crude model.

[2]P-value for the combined test of the different categories: 0.163

[3]Adjusted for smoking, education, alcohol, coffee, total energy intake, body mass index, physical activity, living alone

[4]P-value for the combined test of the different categories: 0.271

[5]Adjusted for smoking, education, alcohol, coffee, total energy intake, body mass index, physical activity, living alone, fruit and vegetables, vitamin and mineral supplements, and milk

[6]P-value for the combined test of the different categories: 0.22

[7]Adjusted for smoking, education, alcohol, coffee, total energy intake, body mass index, physical activity, living alone, fruit and vegetables, vitamin and mineral supplements, aspirin, milk, and high blood pressure

[8]P-value for the combined test of the different categories: 0.221

Table S10. Sensitivity analysis for the association of milk intake, excluding those with missing information on milk intake, with Parkinson's disease among women and men (n=71 765, cases=1124). Missing information on fermented milk were replaced with 0 intake. Other covariates were imputed.

Glasses* per day	Total N	Cases	Model 1[1,2] HR (95% CI)	Model 2[3,4] HR (95% CI)	Model 3[5,6] HR (95% CI)	Model 4[7,8] HR (95% CI)
<0.2	8030	87	Referent	Referent	Referent	Referent
0.2-0.8	14848	224	1.22 (0.95, 1.56)	1.22 (0.95, 1.56)	1.23 (0.96, 1.58)	1.24 (0.96, 1.59)
0.8-1.1	17917	283	1.16 (0.91, 1.47)	1.14 (0.90, 1.46)	1.14 (0.90, 1.46)	1.15 (0.90, 1.46)
1.1-2.0	18206	326	1.25 (0.98, 1.58)	1.25 (0.98, 1.59)	1.24 (0.97, 1.58)	1.24 (0.98, 1.59)
>2.0	12764	204	1.11 (0.86, 1.42)	1.13 (0.87, 1.47)	1.11 (0.85, 1.44)	1.12 (0.86, 1.45)

CI, confidence interval; HR, hazard ratio; N, number

*1 glass = 200 ml.

[1]Adjusted for sex.

[2]P-value for the combined test of the different categories: 0.339.

[3]Adjusted for sex, smoking, education, alcohol, coffee, total energy intake, body mass index, physical activity, living alone.

[4]P-value for the combined test of the different categories: 0.381.

[5]Adjusted for sex, smoking, education, alcohol, coffee, total energy intake, body mass index, physical activity, living alone, fruit and vegetables, vitamin and mineral supplements, yogurt and soured milk.

[6]P-value for the combined test of the different categories: 0.339.

[7]Adjusted for sex, smoking, education, alcohol, coffee, total energy intake, body mass index, physical activity, living alone, fruit and vegetables, vitamin and mineral supplements, aspirin, yogurt and soured milk, and high blood pressure.

[8]P-value for the combined test of the different categories: 0.335.

Table S11. Sensitivity analysis for the association of fermented milk intake, excluding those with missing information on fermented milk intake, with Parkinson's disease among women and men (n=60 010, cases=940). Missing information on milk was replaced with 0 intakes. Other covariates were imputed.

Glasses* per day	Total N	Cases	Model 1[1,2] HR (95% CI)	Model 2[3,4] HR (95% CI)	Model 3[5,6] HR (95% CI)	Model 4[7,8] HR (95% CI)
<0.2	7102	117	Referent	Referent	Referent	Referent
0.2-0.8	16475	218	0.71 (0.57, 0.89)	0.70 (0.56, 0.88)	0.68 (0.54, 0.86)	0.69 (0.55, 0.86)
0.8-1.1	19687	325	0.83 (0.67, 1.03)	0.80 (0.65, 1.00)	0.80 (0.64, 0.99)	0.80 (0.64, 0.99)
>1.1	16746	280	0.82 (0.66, 1.01)	0.79 (0.64, 0.99)	0.79 (0.63, 0.98)	0.79 (0.63, 0.99)

CI, confidence interval; HR, hazard ratio; N, number

*1 glass = 200 ml.

[1]Adjusted for sex.

[2] P-value for the combined test of the different categories: 0.031.

[3]Adjusted for sex, smoking, education, alcohol, coffee, total energy intake, body mass index, physical activity, living alone.

[4] P-value for the combined test of the different categories: 0.027.

[5]Adjusted for sex, smoking, education, alcohol, coffee, total energy intake, body mass index, physical activity, living alone, fruit and vegetables, vitamin- and mineral supplements, and milk.

[6] P-value for the combined test of the different categories: 0.013.

[7]Adjusted for sex, smoking, education, alcohol, coffee, total energy intake, body mass index, physical activity, living alone, fruit and vegetables, vitamin and mineral supplements, aspirin, milk, and high blood pressure.

[8] P-value for the combined test of the different categories: 0.014.

Table S12. Sensitivity analysis, excluding the first three years of follow-up, for the association of milk intake with Parkinson's disease among women and men

(n=79 843, cases n=1174).

Glasses* per day	Total N	Cases	Model 1[1,2] HR (95% CI)	Model 2[3,4] HR (95% CI)	Model 3[5,6] HR (95% CI)	Model 4[7,8] HR (95% CI)
<0.2	17807	204	Referent	Referent	Referent	Referent
0.2-0.8	14603	211	1.26 (1.04, 1.53)	1.24 (1.02, 1.50)	1.26 (1.04, 1.54)	1.27 (1.04, 1.54)
0.8-1.1	17451	263	1.22 (1.01, 1.46)	1.18 (0.98, 1.42)	1.19 (0.99, 1.43)	1.19 (0.99, 1.43)
1.1-2.0	17640	304	1.33 (1.12, 1.59)	1.30 (1.09, 1.56)	1.30 (1.09, 1.56)	1.31 (1.09, 1.57)
>2.0	12342	192	1.19 (0.97, 1.45)	1.19 (0.97, 1.47)	1.18 (0.96, 1.45)	1.18 (0.96, 1.45)

CI, confidence interval; HR, hazard ratio; N, number

*1 glass = 200 ml.

[1]Adjusted for a sex
[2]P-value for the combined test of the different categories: 0.03
[3]Adjusted for sex, smoking, education, alcohol, coffee, total energy intake, body mass index, physical activity, living alone
[4]P-value for the combined test of the different categories: 0.066
[5]Adjusted for sex, smoking, education, alcohol, coffee, total energy intake, body mass index, physical activity, living alone, fruit and vegetables, vitamin and mineral supplements, yogurt and soured milk
[6]P-value for the combined test of the different categories: 0.056
[7]Adjusted for sex, smoking, education, alcohol, coffee, total energy intake, body mass index, physical activity, living alone, fruit and vegetables, vitamin and mineral supplements, aspirin, yogurt and soured milk, and high blood pressure
[8]P-value for the combined test of the different categories: 0.055

Table S13. Sensitivity analysis, excluding the first three years of follow-up, for the association of fermented milk intake with Parkinson's disease among women and men (n=79 843, cases=1174).

Glasses* per day	Total N	Cases	Model 1[1,2] HR (95% CI)	Model 2[3,4] HR (95% CI)	Model 3[5,6] HR (95% CI)	Model 4[7,8] HR (95% CI)
<0.2	28128	397	Referent	Referent	Referent	Referent
0.2-0.8	16164	208	0.95 (0.81, 1.13)	0.93 (0.79, 1.10)	0.92 (0.78, 1.09)	0.92 (0.78, 1.10)
0.8-1.1	19244	307	1.12 (0.97, 1.31)	1.08 (0.93, 1.26)	1.10 (0.94, 1.28)	1.10 (0.94, 1.29)
>1.1	16307	262	1.10 (0.94, 1.28)	1.08 (0.92, 1.27)	1.09 (0.93, 1.28)	1.09 (0.93, 1.28)

CI, confidence interval; HR, hazard ratio; N, number

*1 glass = 200 ml.

[1]Adjusted for sex.

[2] P-value for the combined test of the different categories: 0.2.

[3]Adjusted for sex, smoking, education, alcohol, coffee, total energy intake, body mass index, physical activity, living alone

[4] P-value for the combined test of the different categories: 0.296.

[5]Adjusted for sex, smoking, education, alcohol, coffee, total energy intake, body mass index, physical activity, living alone, fruit and vegetables, vitamin- and mineral supplements, and milk.

[6] P-value for the combined test of the different categories: 0.187.

[7]Adjusted for sex, smoking, education, alcohol, coffee, total energy intake, body mass index, physical activity, living alone, fruit and vegetables, vitamin and mineral supplements, aspirin, milk, and high blood pressure.

[8] P-value for the combined test of the different categories: 0.189.

Table S14. Association between milk intake and Parkinson's disease among women with time updated information on milk intake in 1987-89 (n= 61424, cases n=540, 1405972 person-years of follow-up).

Glasses* per day	Model 1[1,2] HR (95 % CI)	Model 2[3,4] HR (95 % CI)	Model 3[5,6] HR (95 % CI)	Model 4[7,8] HR (95 % CI)	Model 5[9,10] HR (95 % CI)
<0.2	Referent	Referent	Referent	Referent	Referent
0.2-0.7	1.44 (1.09, 1.91)	1.38 (1.04, 1.83)	1.40 (1.06, 1.86)	1.40 (1.06, 1.86)	1.40 (1.06, 1.86)
0.8-1.0	1.11 (0.85, 1.44)	1.09 (0.83, 1.42)	1.10 (0.84, 1.43)	1.11 (0.85, 1.44)	1.10 (0.84, 1.43)
1.1-2.0	1.49 (1.14, 1.94)	1.44 (1.10, 1.90)	1.43 (1.09, 1.88)	1.44 (1.09, 1.90)	1.43 (1.09, 1.89)
>2.0	1.18 (0.87, 1.60)	1.17 (0.86, 1.60)	1.16 (0.85, 1.59)	1.18 (0.86, 1.62)	1.16 (0.85, 1.59)

CI, confidence interval; HR, hazard ratio

[*]1 glass =200 ml
[1]Crude model
[2]P-value for the combined test of the different categories: 0.014
[3]Adjusted for smoking, education, alcohol, coffee, total energy intake, body mass index, physical activity, living alone
[4]P-value for the combined test of the different categories: 0.031
[5]Adjusted for smoking, education, alcohol, coffee, total energy intake, body mass index, physical activity, living alone, fruit and vegetables, vitamin and mineral supplements, yogurt and soured milk
[6]P-value for the combined test of the different categories: 0.035
[7]Adjusted for smoking, education, alcohol, coffee, total energy intake, body mass index, physical activity, living alone, fruit and vegetables, vitamin and mineral supplements, aspirin, yogurt and soured milk, and high blood pressure
[8]P-value for the combined test of the different categories: 0.036
[9]Adjusted for smoking, education, alcohol, coffee, total energy intake, body mass index, physical activity, living alone, fruit and vegetables, vitamin and mineral supplements, aspirin, yogurt and soured milk, high blood pressure and hormone replacement
[10]P-value for the combined test of the different categories: 0.035

Table S15. Association between fermented milk intake and Parkinson's disease among women with time updated information on fermented milk intake in 1987-89 (n= 61424, cases n=540, 1405972 person-years of follow-up).

Glasses* per day	Model 1[1,2] HR (95 % CI)	Model 2[3,4] HR (95 % CI)	Model 3[5,6] HR (95 % CI)	Model 4[7,8] HR (95 % CI)	Model 5[9,10] HR (95 % CI)
<0.2	Referent	Referent	Referent	Referent	Referent
0.2-0.8	1.06 (0.83, 1.35)	1.00 (0.78, 1.28)	0.97 (0.76, 1.25)	0.97 (0.76, 1.25)	0.97 (0.76, 1.24)
0.8-1.1	1.08 (0.87, 1.36)	0.97 (0.77, 1.22)	0.98 (0.78, 1.24)	0.98 (0.78, 1.24)	0.97 (0.77, 1.23)
>1.1	1.45 (1.14, 1.84)	1.27 (0.98, 1.63)	1.26 (0.98, 1.63)	1.26 (0.97, 1.62)	1.25 (0.96, 1.61)

CI, confidence interval; HR, hazard ratio

*1 glass =200 ml

[1]Crude model

[2] P-value for the combined test of the different categories: 0.017

[3]Adjusted for smoking, education, alcohol, coffee, total energy intake, body mass index, physical activity, living alone

[4] P-value for the combined test of the different categories: 0.148

[5]Adjusted for smoking, education, alcohol, coffee, total energy intake, body mass index, physical activity, living alone, fruit and vegetables, vitamin- and mineral supplements, and milk.

[6] P-value for the combined test of the different categories: 0.161

[7]Adjusted for smoking, education, alcohol, coffee, total energy intake, body mass index, physical activity, living alone, fruit and vegetables, vitamin and mineral supplements, aspirin, milk, and high blood pressure.

[8] P-value for the combined test of the different categories: 0.171

[9]Adjusted for smoking, education, alcohol, coffee, total energy intake, body mass index, physical activity, living alone, fruit and vegetables, vitamin and mineral supplements, aspirin, milk, high blood pressure and hormone replacement.

[10]P-value for the combined test of the different categories: 0.184
